# The FTO-CMPK2 Pathway in Fibroblast-like Synoviocytes Modulates Rheumatoid Arthritis Synovial Inflammation and Cartilage Homeostasis via mtDNA Regulation

**DOI:** 10.7150/ijbs.90677

**Published:** 2024-02-11

**Authors:** Li Jin, Qiyue Chen, Ke Hu, Dandan Fan, Heping Zhang, Jiaxin Deng, Weizhong Qi, Qinghong Yu

**Affiliations:** 1 Rheumatology and Clinical Immunology, ZhuJiang Hospital, Southern Medical University, 510280, Guangzhou, China.; 2 Translational Medicine Research Center, ZhuJiang Hospital, Southern Medical University, 510280, Guangzhou, China.; 3 Clinical Research Center, ZhuJiang Hospital, Southern Medical University, 510280, Guangzhou, China.

**Keywords:** rheumatoid arthritis, FTO, CMPK2, synovitis, Mitochondrial DNA

## Abstract

In rheumatoid arthritis (RA), a debilitating autoimmune disorder marked by chronic synovial inflammation and progressive cartilage degradation, fibroblast-like synoviocytes (FLS) are key pathogenic players. Current treatments targeting these cells are limited. Our study focused on the Fat Mass and Obesity-associated protein (FTO), known for its roles in cell proliferation and inflammatory response modulation, and its involvement in RA. We specifically examined the inflammatory regulatory roles of FTO and CMPK2, a mitochondrial DNA synthesis protein, in FLS. Utilizing a combination of in vitro and in vivo methods, including FTO inhibition and gene knockdown, we aimed to understand FTO's influence on RA progression and chondrocyte functionality. Our findings showed that increased FTO expression in RA synovial cells enhanced their proliferation and migration and decreased senescence and apoptosis. Inhibiting FTO significantly slowed the disease progression in our models. Our research also highlighted that the FTO-CMPK2 pathway plays a crucial role in regulating synovial inflammation through the mtDNA-mediated cGAS/STING pathway, affecting chondrocyte homeostasis. This study indicates that targeting the FTO-CMPK2 axis could be a promising new therapeutic strategy for managing RA.

## Introduction

Rheumatoid arthritis (RA) is a persistent autoimmune inflammatory condition predominantly marked by inflammation of synovial tissues and consequential joint deterioration. Rheumatoid arthritis fibroblast-like synoviocytes (RA-FLS) are pivotal pathogenic cells in RA, contributing to the sustained inflammation of synovial tissue and the progressive erosion of articular cartilage. The invasiveness and pro-inflammatory properties of RA-FLS constitute primary factors driving synovial inflammation and articular cartilage damage during the course of RA progression^1^. Despite certain clinical advancements in RA management, there is currently no definitive intervention available to address the sustained synovial inflammation and joint tissue damage instigated by RA-FLS^2^. Consequently, a thorough investigation into the pathogenic mechanisms of RA-FLS presents a potential therapeutic avenue for mitigating synovial inflammation and safeguarding cartilage, thereby enhancing the well-being of individuals suffering from RA and preventing disease progression.

Recent research has unveiled the significant role of Fat mass and obesity-associated protein (FTO) in various diseases. As the pioneering demethylase to be discovered, FTO is instrumental in modulating a wide spectrum of physiological and pathological processes, including adipogenesis, tumorigenesis, aging, and metabolism^3^. Studies have indicated that reduced FTO gene expression within peripheral blood mononuclear cells is a potential contributing factor to RA^4^. However, the specific role of FTO in RA-FLS and its biological behavior in regulation remain enigmatic. Consequently, this study aims to clarify the mechanistic contribution of FTO to the progression of RA.

Mitochondria are complex intracellular organelles with multifarious vital functions. Mitochondrial DNA (mtDNA), as the genetic material of cellular mitochondria, not only regulates energy metabolism but also serves as a damage-associated molecular pattern (DAMP), inciting inflammation and immune responses. The maintenance of mitochondrial homeostasis in RA-FLS closely correlates with RA development^5^, ^6^. Previous investigations have unearthed a notable augmentation in mtDNA within RA synovium, and cytoplasmic mtDNA can activate inflammatory pathways, including the cGAS/STING axis, which plays a pivotal role in RA^7^. Nevertheless, the incipient and propelling mechanisms of mtDNA in RA-FLS remain elusive.

In this study, our objective was to probe into the functional role of FTO in the initiation and progression of RA. We observed heightened FTO expression in knee joint synovium samples from both RA patients and adjuvant-induced arthritis (AIA) mice. Leveraging mRNA sequencing data subsequent to FTO knockdown, we substantiated that FTO regulates mitochondrial mtDNA-mediated inflammation through CMPK2. Suppression of FTO or CMPK2 markedly ameliorated synovial inflammation and cartilage damage, thus mitigating the advancement of RA. In summary, our research underscores the potential of FTO-CMPK2 as a promising novel therapeutic target for RA. This study provides invaluable insights into comprehending the pathophysiological underpinnings of RA and advancing novel therapeutic strategies for this debilitating condition.

## Methods

### Tissue samples

Synovial and cartilage tissue samples were acquired from Zhujiang Hospital, Southern Medical University. The study included RA patients who had undergone knee joint replacement surgery, as well as patients with osteoarthritis (OA). RA patients (n=5) were selected based on meeting the 2010 diagnostic criteria of the American College of Rheumatology (ACR) and the European League Against Rheumatism (EULAR) for RA, while the control group comprised OA patients (n=5). Careful matching of gender and age was carried out between RA and OA patients to mitigate potential gender and age-related biases. The research protocol received approval from the Ethics Committee of Zhujiang Hospital, Southern Medical University (Ethics No. 2022-ky-165-02), and all surgical patients provided informed consent. Clinical specimens of synovial and cartilage tissues were collected for subsequent immunohistochemistry and immunofluorescence experiments.

### Cell culture

Human rheumatoid arthritis fibroblast-like synoviocytes (RA-FLS) were procured from Cell Applications and cultured in DMEM supplemented with 10% fetal calf serum, 100 U/ml penicillin, and 100 μg/ml streptomycin. These cells were incubated at 37°C in an atmosphere with 5% CO2, and the culture medium was refreshed every 2-3 days. Upon reaching 90% confluence, the cells were subcultured using EDTA-trypsin and subsequently employed in further experiments.

### Small RNAi transfection

Small interfering RNAs (siRNAs) directed against the human FTO gene and a negative control (Scr siRNA) were obtained from RiboBio Co., Ltd. (Guangzhou, China). RA-FLS cells were seeded in six-well plates prior to transfection. Transfection was carried out using LipofectamineTM 3000 reagent (L3000015) in accordance with the manufacturer's instructions. Following a 4-6 hour incubation, the culture medium was replaced with fresh medium, and a series of assays were conducted on the cells.

### Real-time quantitative PCR

Total RNA was extracted from cells using the Trizol method, followed by quantification using a NanoDrop 2000 spectrophotometer. Subsequently, complementary DNA (cDNA) was synthesized using 5X PrimeScript RT Master Mix. Real-time quantitative PCR (qPCR) was performed using ChamQ SYBR qPCR Master Mix (Vazyme) and conducted on an ABI system. The expression levels of all genes listed in Table 1 were determined relative to the expression of the glyceraldehyde-3-phosphate dehydrogenase (GAPDH) gene, which served as a reference for normalization.

### Western blot

Proteins were extracted from both cell and tissue using RIPA lysis buffer supplemented with protease inhibitors. Protein concentrations were determined through the utilization of a BCA assay kit. Subsequently, equivalent amounts of protein were resolved on SDS-PAGE gels and subsequently transferred onto PVDF membranes. After blocking with 5% milk at room temperature for 2 hours, the membranes underwent overnight incubation at 4°C with primary antibodies. This was followed by three washes with Tween 20 Tris-buffered saline. Secondary antibodies were applied at a 1:1000 dilution and incubated at room temperature for 2 hours. Protein bands were detected using an enhanced chemiluminescence kit from Thermo Scientific, USA, and protein expression levels were quantified using ImageJ software.

### RNA sequencing (RNA-seq)

Total RNA from control and FTO knockdown groups was extracted using Trizol. Poly-A-tailed RNA libraries were constructed, and quality control was performed using the Bioptic Qseq100 platform. Sequencing was carried out using the Illumina high-throughput sequencing platform (NovaSeq 6000).

### Flow cytometry

Apoptosis was evaluated following the manufacturer's protocol with the Annexin V apoptosis detection kit. Cells from different treatment groups were harvested, trypsinized (without EDTA), centrifuged, and suspended in 300 μL of binding buffer. Subsequently, 5 μL of Annexin V-FITC and 5 μL of propidium iodide (PI) were added and gently mixed into the cell suspension. The cells were then incubated in the dark at room temperature for 15 minutes before undergoing apoptosis analysis via flow cytometry.

### β-Galactosidase staining

β-galactosidase staining was carried out following the manufacturer's instructions. Cells from various treatment groups were seeded in six-well plates, washed with PBS 1-2 times, and then fixed with 1 mL of β-galactosidase fixative at room temperature for 15 minutes. Following three washes with PBS, 1 mL of staining working solution was added, and the cells were incubated at 37°C overnight. Images were acquired using a standard optical microscope.

### EdU (5-ethynyl-2'-deoxyuridine) assay

Cells from the various treatment groups were incubated with 10% FBS-diluted EdU to a final concentration of 10 μmol/L for 4 hours. Following the removal of the culture medium, the cells were rinsed twice with PBS and subsequently fixed using 4% paraformaldehyde, and subjected to Apollo staining. Cell proliferation was assessed using a fluorescence microscope.

### Transwell assay

RA-FLS cells transfected with si-FTO were enzymatically detached with trypsin and adjusted to a cell density of approximately 1 × 10^4 cells. Subsequently, 200 μL of this cell suspension was introduced into the upper compartment of a Transwell insert, while the lower compartment was loaded with 600 μL of medium containing 10% FBS. The cells were then cultivated in a 37°C incubator for 12 hours, after which they were fixed with 4% paraformaldehyde, stained with crystal violet for a duration of 10-15 minutes, and finally enumerated under a microscope.

### Cell immunofluorescence

Cells from different treatment groups were seeded onto coverslips, allowed to attach overnight, fixed with 4% paraformaldehyde, rinsed with PBS, permeabilized using Triton X-100 for 10 minutes, and then blocked with goat serum for 1 hour. Primary antibodies were diluted as per the manufacturer's instructions and incubated overnight at 4°C. After three PBS washes, cells were exposed to corresponding fluorescent secondary antibodies at room temperature for 1 hour. Following additional PBS washes, coverslips were mounted using a mounting medium containing DAPI, and images were captured using laser confocal microscopy.

### Cell proliferation assay using CCK-8

RA-FLS cells subjected to different treatments were seeded at an appropriate density into 96-well plates. After 48 hours of treatment with various concentrations of FB23, in each well, 100 μL of CCK-8 solution was introduced, and the plate was then incubated for 4 hours. The absorbance (A) values were subsequently determined at a wavelength of 450 nm utilizing a microplate reader. Cell viability after drug treatment was calculated.

### Construction of animal synovitis model and therapeutic intervention

Eight-week-old male C57/BL6 mice, weighing 20 ± 1.3 g, were obtained from the Southern Medical University Animal Center, with six mice in each group. AIA model was established. On day 0, complete Freund's adjuvant was subcutaneously injected into the groin to initiate immune induction. On day 14, incomplete Freund's adjuvant was injected into the knee joint cavity to induce delayed hypersensitivity reactions. Prior to all procedures, mice were anesthetized using the mouse-specific anesthetic isoflurane for inhalation anesthesia. On day 21, mice were treated with intraperitoneal injections of FB23 (10mg/kg) or TNF-α neutralizing antibodies (20 µg/mL), and intra-articular injection of CMPK2 knockdown adenovirus as per treatment requirements, once a week. Animal research was reviewed and approved by the Ethics Committee for Animal Care and Use at Southern Medical University Zhujiang Hospital (NO. LAEC-2021-129FS).

### Tissue section preparation and histological staining

The collected synovial and cartilage tissues were immersed in 4% paraformaldehyde for a 24-hour fixation period. Following decalcification, the tissues were embedded in paraffin and then sectioned into 5 μm thick slices. Subsequently, histological staining was conducted using hematoxylin and eosin (H&E) staining reagents as well as toluidine blue (TB) staining reagents.

### Immunofluorescence immunohistochemistry

Immunohistochemistry and immunofluorescence staining were conducted on 5 μm-thick tissue sections embedded in paraffin. These sections underwent a series of preparatory steps, including dehydration, deparaffinization, and rehydration. Antigen retrieval was performed, followed by blocking with goat serum. The tissue sections were then subjected to an overnight incubation with specific antibodies and subsequently treated with secondary antibodies. Afterward, the sections were examined under a microscope, and suitable fields were chosen for imaging.

### Antibody and product number

Anti-KI67 (abcam, ab16667), Anti-KI67 (ab279653), Anti-TOMM20 (abcam, ab186735), Anti-FTO (ab126605), Anti-Vimentin (Santa, sc-80975), Anti-COL II (abcam, ab34712), Anti-MMP3 (abcam, ab52915), Anti-MMP13 (abcam, ab39012), Anti-dsDNA (abcam, ab27156), Anti-CMPK2 (Proteintech, 25877-1-AP), Anti-STING (CST.13647), Anti-cGAS (Proteintech, 29958-1-AP), Anti-TBK1 (abcam, ab40676), Anti-p-TBK1 (CST.5483), Anti-p-IRF3 (CST.29057), Anti-IL-1β (abcam, ab315084), Anti-IL-6 (abcam, ab290735), Anti-GADPH (abcam, ab8245).

### ELISA assay

Murine serum was collected to measure cytokine detection. ELISA kits used included those specific for IL-1β, IL-6. For specific steps, follow the instructions recommended by the reagent manufacturer. The optical density (OD) value for each sample was determined at 450 nm.

### Synovitis score

The Arthritis Synovial Score was determined based on the assessment of three chronic synovitis characteristics: The assessment included the evaluation of three specific characteristics: the expansion of the lining cell layer, the density of synovial stroma cells, and the extent of leukocytic infiltrate. Each of these attributes was graded on a semi-quantitative scale ranging from 0 (indicating absence) to 3 (indicating strong presence), with separate grading conducted for each characteristic. The overall score was determined by summing the individual scores for these features and was interpreted as follows: scores of 0-1 indicated the absence of synovitis, scores of 2-4 signified the presence of low-grade synovitis, and scores of 5-9 indicated the presence of high-grade synovitis^8^.

### Statistical analysis

All reported values are expressed as the mean ± standard deviation (SD), derived from a minimum of three independent experiments. Data analysis was carried out using either paired t-tests or analysis of variance (ANOVA) with GraphPad Prism software. Statistical significance was defined as P < 0.05.

## Results

### Increased expression of FTO in fibroblastic synovial cells in patients with RA

**Figure 1:**
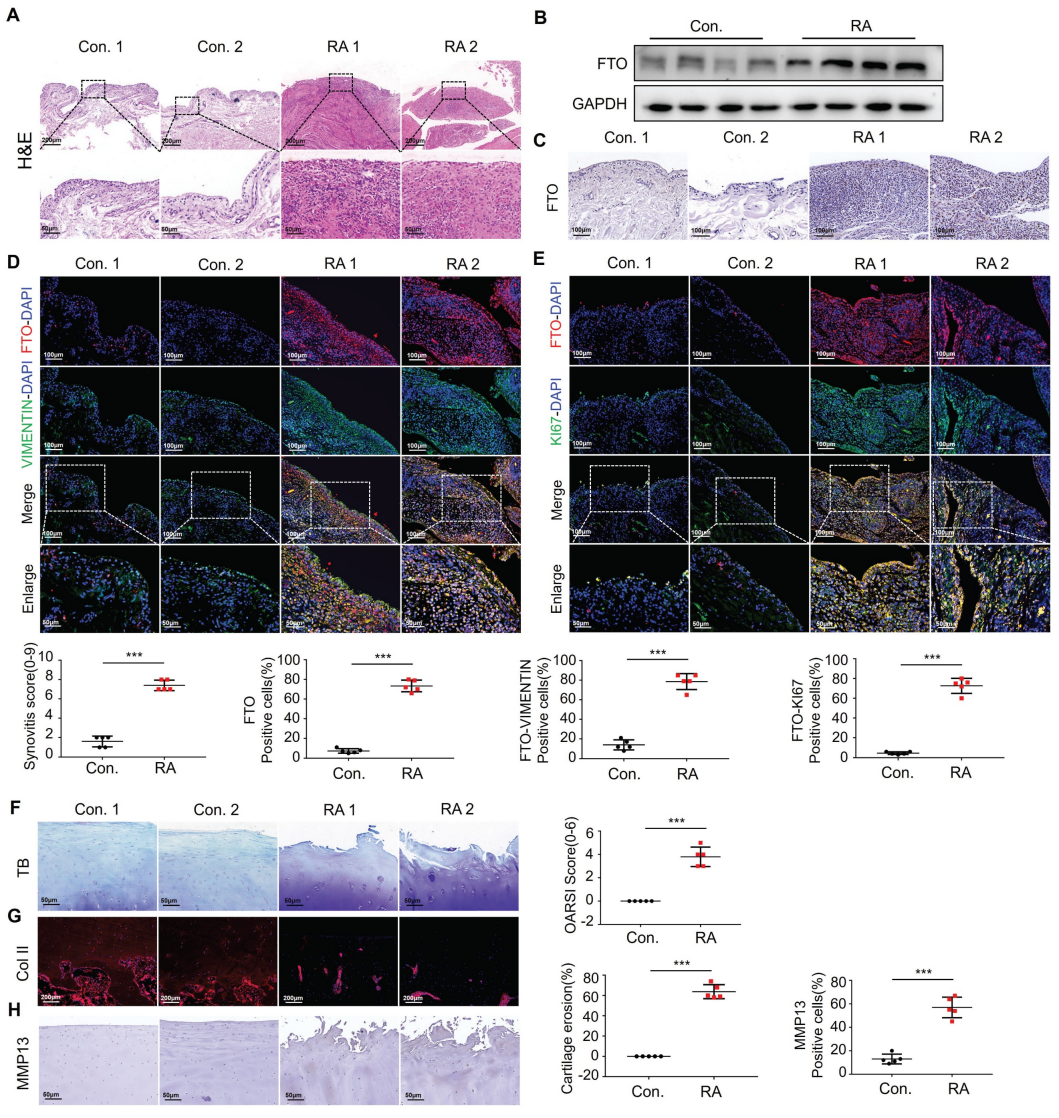
** Increased expression of FTO in FLS in patients with RA.** (A) H&E staining analysis of synovial tissue from control and RA patients (Scale bars, 200 μm or 50 μm). (B) Western blotting to detect FTO expression levels in synovial tissue. (C) Immunohistochemistry for assessing FTO expression (Scale bars, 100 μm). (D) Immunofluorescence dual staining for FTO and VIMENTIN (Scale bars, 100 μm or 50 μm). (E) Immunofluorescence dual staining for FTO and KI67 (Scale bars, 100 μm or 50 μm). (F) Toluidine blue staining analysis of cartilage damage in control and RA patients (Scale bars, 50 μm). (G) Immunofluorescence analysis of cartilage COII expression (Scale bars, 200 μm). (H) Immunohistochemistry demonstrating cartilage MMP13 expression (Scale bars, 50 μm). Data are shown as mean ± SD (n = 5). **P < 0.05, ***P < 0.01. ns > 0.05.

To assess the histopathological characteristics of human synovial tissue samples, we procured synovial tissues from patients diagnosed with RA and OA as a control group through clinical surgical procedures. We employed H&E staining and immunohistochemical staining for pathological examination. When comparing the RA group to the control group, it was apparent that there was pronounced synovial hyperplasia and a high degree of cellular infiltration, leading to increased scores for synovial inflammation (Figure 1A). To further explore the involvement of FTO in the development of RA, we conducted a thorough assessment of FTO expression in human RA synovial tissues. Initially, protein validation of synovial tissues was performed, revealing a substantial increase in FTO expression within the RA synovium When contrasted with the control group (Figure 1B). Concurrently, the results of immunohistochemical staining revealed an increase in FTO expression within the synovial tissues of RA patients as opposed to the control group (Figure 1C).

Using VIMENTIN to label synovial tissues and FTO to label cells, immunofluorescence double staining (FTO-VIMENTIN) unveiled a greater prevalence of cells displaying positive FTO expression within the synovial tissues of RA patients (Figure 1D). Subsequently, we conducted FTO-KI67 double fluorescence staining on synovial cells, revealing a marked proliferative capacity among FTO-positive synovial cells within the RA patient synovial tissues (Figure 1E).

In the context of RA progression, cartilage damage constitutes a pivotal and destructive joint manifestation^9^, ^10^. Thus, we meticulously examined the extent of cartilage damage in RA patients. Utilizing Toluidine Blue staining, we detected significant cartilage layer damage in RA patients, characterized by the loss of transparent cartilage, leaving behind only hypertrophic deep-layer chondrocytes (Figure 1F). Immunofluorescence staining for COL II indicated conspicuous cartilage tissue degradation in RA patients, accompanied by a reduction in collagen content (Figure 1G). Immunohistochemical staining further corroborated these findings by demonstrating an elevation in MMP-13 levels, suggestive of extensive chondrocyte damage and extracellular matrix degradation (Figure 1H).

### Heightened FTO expression in AIA mice synovial cells

**Figure 2:**
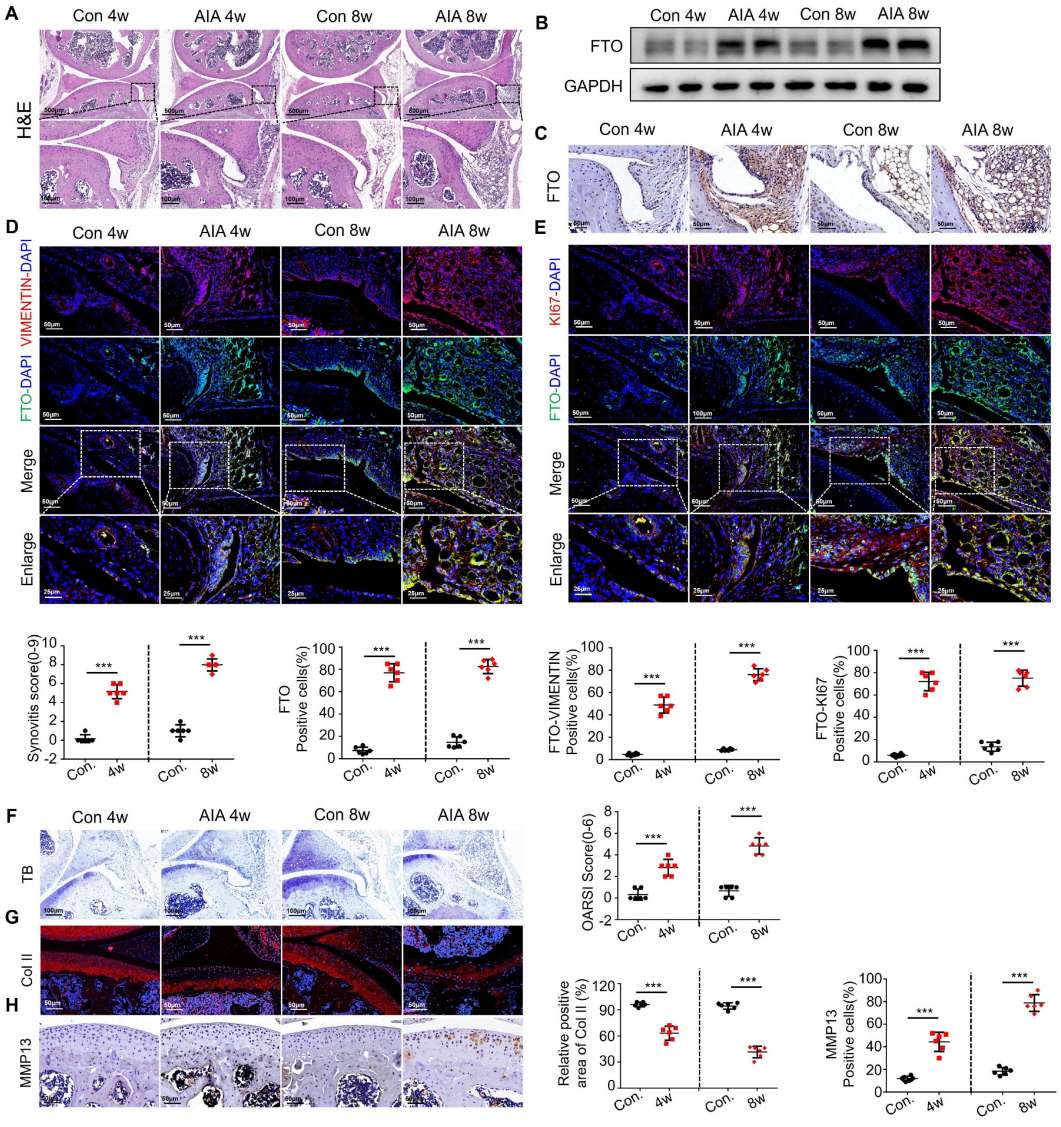
** Heightened FTO expression in AIA mice synovial cells.** (A) H&E staining analysis of mouse synovial tissue (Scale bars, 500 or 100 μm). (B) Western blotting to detect FTO expression levels in mouse synovial tissue. (C) Immunohistochemistry for assessing FTO expression (Scale bars, 50 μm). (D) Immunofluorescence dual staining for FTO and VIMENTIN in mouse synovial tissue (Scale bars, 50 μm or 25 μm). (E) Immunofluorescence dual staining for FTO and KI67 in mouse synovial tissue (Scale bars, 50 μm or 25 μm). (F) Toluidine blue staining analysis of cartilage damage in control and AIA mice (Scale bars, 100 μm). (G) Immunofluorescence analysis of cartilage COII expression (Scale bars, 50 μm). (H) Immunohistochemistry demonstrating cartilage MMP13 expression (Scale bars, 50 μm). Data are shown as mean ± SD (n = 6). **P < 0.05, ***P < 0.01. ns > 0.05.

In order to conduct an in-depth investigation into the role of FTO in the progression of synovial inflammation in RA, we established murine models using either complete or incomplete Freund's adjuvant, aimed at simulating the pathological processes seen in RA. Histological examination performed through HE staining revealed pronounced synovial inflammation at both the 4-week and 8-week time points. This inflammation was characterized by marked synovial hyperplasia and robust cellular infiltration, ultimately resulting in a significant increase in synovitis scores in the AIA mice (Figure 2A). Western blot analysis unequivocally revealed a significant increase in FTO expression within the synovial tissues of the AIA mice (Figure 2B), a finding that was consistently supported by immunohistochemistry (Figure 2C).

Subsequently, we sought to delineate the spatial distribution of FTO within the synovial tissues of AIA mice, employing VIMENTIN-FTO co-labeling techniques. This analysis revealed a heightened presence of FTO-positive synovial cells in the synovial tissues of AIA mice (Figure 2D). To further elucidate the impact of FTO on cellular proliferation, we conducted dual immunofluorescence staining for FTO and KI67 in synovial cells within the RA mouse model, where we observed a pronounced proliferative capacity among FTO-positive synovial cells (Figure 2E).

Continuing our investigation, we assessed the extent of damage in mouse articular cartilage tissues. Toluidine blue staining unveiled discernible cartilage loss within the articular cartilage layer of AIA mice, characterized by surface cartilage erosion and the presence of enlarged chondrocytes (Figure 2F). Immunofluorescence staining for COL II corroborated these findings, showcasing a significant degradation of collagen in the articular cartilage tissues of AIA mice (Figure 2G). Furthermore, immunohistochemistry highlighted an elevated expression of MMP13, suggesting an aberrant matrix degradation process in the cartilage cells of AIA mice (Figure 2H).

### FTO is involved in regulating mtDNA-mediated synovitis

**Figure 3:**
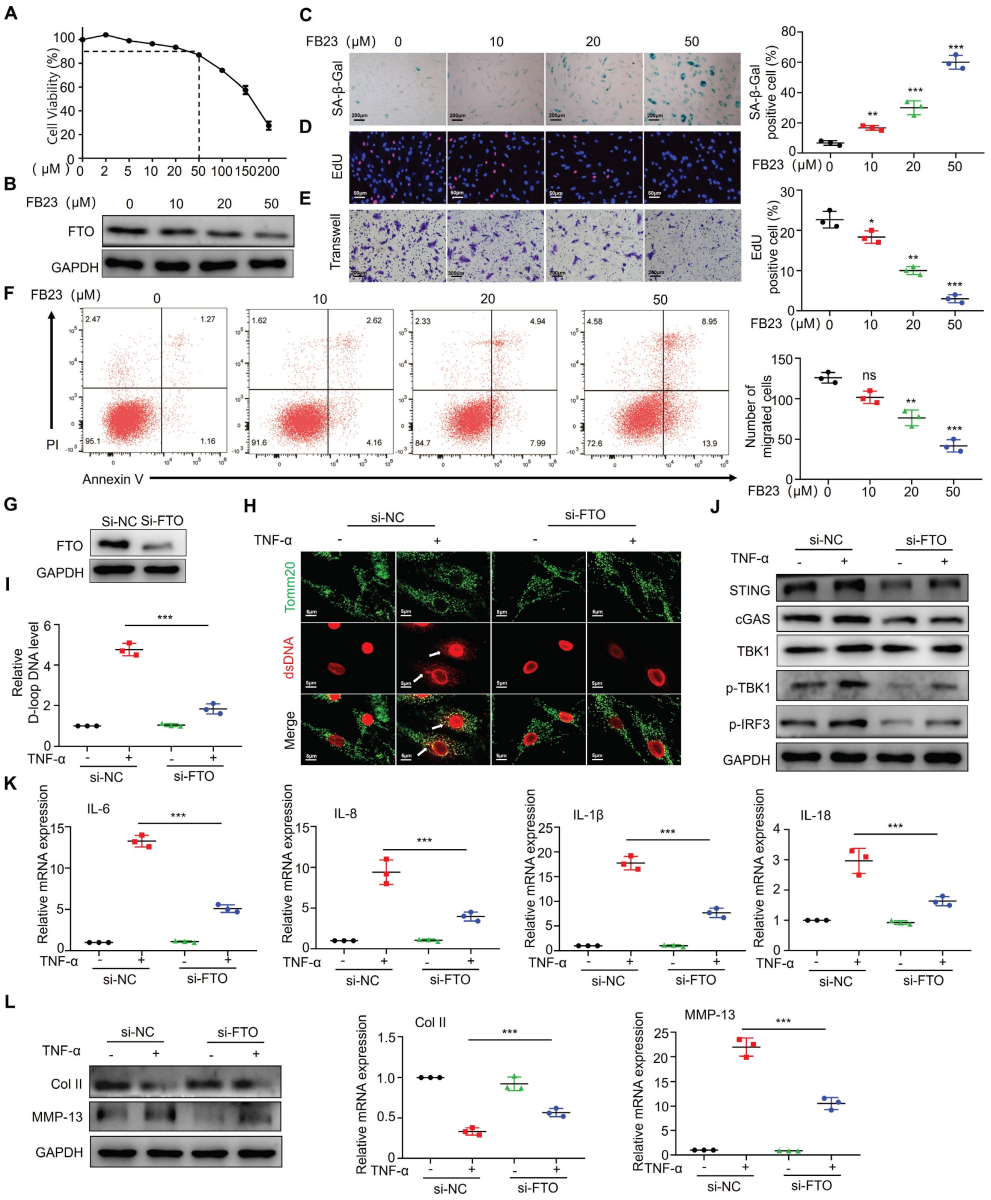
** FTO is involved in regulating mtDNA-mediated synovitis.** (A) Cell viability curve of RA-FLS treated with different concentrations of FB23. (B) Western blotting to assess FTO expression in RA-FLS treated with different concentrations of FB23. (C) β-galactosidase assay to evaluate the impact of FB23 at different concentrations on RA-FLS senescence (Scale bars, 200 μm). (D) EdU assay examining the influence of FB23 at different concentrations on RA-FLS proliferation (Scale bars, 50 μm). (E) Transwell assay to assess the effect of FB23 at different concentrations on RA-FLS migration (Scale bars, 200 μm). (F) Flow cytometry analysis of the impact of FB23 at different concentrations on RA-FLS apoptosis. (G) Efficiency of FTO knockdown determined by Western blotting. (H) Immunofluorescence detecting ds-DNA and Tomm20 expression in RA-FLS cells (Scale bars, 5 μm). (I) D-loop DNA expression levels. (J) Assessment of cGAS/STING pathway expression and activation by Western blotting and qPCR. (K) qPCR analysis of cytokine expression. (L) Western blotting and qPCR analysis of cartilage COL II and MMP13 expression. Data are shown as mean ± SD (n = 3). **P < 0.05, ***P < 0.01. ns > 0.05.

After observing the significant overexpression of FTO in RA patients and mouse synovial tissues, we proceeded to validate the impact of FTO on synovial tissues. Firstly, we conducted drug inhibition studies using the FTO inhibitor FB23. Cell viability of RA-FLS was assessed using CCK8, and the results demonstrated a significant reduction in cell survival at concentrations exceeding 100 uM FB23 (Figure 3A). Therefore, we selected four different concentrations (0, 10, 20, and 50 uM) for subsequent experiments. Western blot analysis revealed that FB23 could markedly inhibit FTO expression in RA-FLS (Figure 3B). Subsequently, we evaluated various aspects of RA-FLS behavior, including aging, proliferation, migration, and apoptosis. The response of RA-FLS to FB23 exhibited a concentration-dependent pattern, with higher FB23 concentrations leading to noticeable promotion of aging, suppression of proliferation, decreased migration ability, and altered apoptosis (Figure 3C-3F). Furthermore, we constructed siRNAs to knock down FTO, intending to observe cellular changes upon FTO depletion. Western blot validation confirmed successful FTO knockdown (Figure 3G). Given that RA is an inflammatory disease, and previous research has highlighted the significance of TNF-α in RA, especially its role in regulating mitochondrial dynamics, we conducted experiments involving TNF-α after FTO knockdown in RA-FLS. Immunofluorescence observations indicated that in the absence of FTO knockdown, TNF-α significantly induced mtDNA expression in RA-FLS, whereas FTO knockdown resulted in reduced mtDNA expression upon TNF-α stimulation (Figure 3H). D-loop analysis, a marker for mtDNA, further confirmed that FTO knockdown reduced the mtDNA synthesis ability induced by TNF-α (Figure 3I).

We postulated that FTO might exert its effects through the mtDNA pathway. To explore this further, we conducted assessments of the common mtDNA-dependent pathway, the cGAS-STING pathway. Detection revealed that in the absence of FTO knockdown, TNF-α could induce the activation of cGAS-STING, whereas FTO knockdown resisted TNF-α-induced cGAS-STING activity (Figure 3J). Concurrently, we examined the expression of common downstream cytokines of the cGAS-STING pathway and found that IL-6, IL-8, IL-1β, and IL-18 were downregulated after FTO knockdown (Figure 3K).

Finally, we co-cultured the supernatant from FTO-knockdown synovial cells with chondrocytes and, through Western blot and qPCR analyses, observed no significant reduction in collagen type II levels and no significant increase in MMP13 levels when TNF-α was added (Figure 3L). This suggests that FTO knockdown in RA-FLS can mitigate inflammatory factor-mediated cartilage damage induced by TNF-α.

### Inhibition of FTO alleviates AIA mouse arthritis

**Figure 4:**
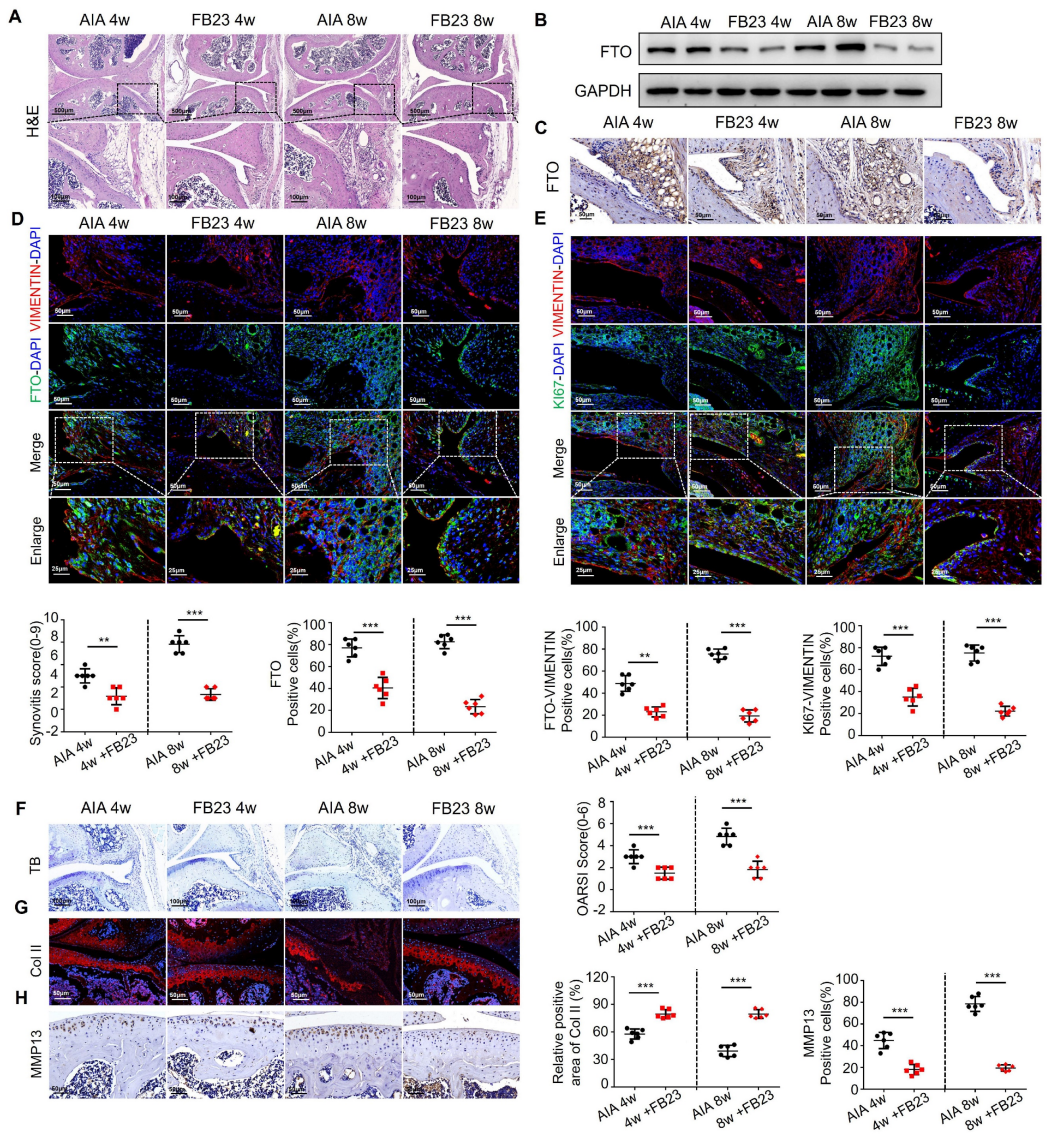
** Inhibition of FTO alleviates AIA mouse arthritis.** (A) H&E staining analysis of mouse synovial tissue (Scale bars, 500 or 100 μm). (B) Western blotting to detect FTO expression levels in mouse synovial tissue. (C) Immunohistochemistry for assessing FTO expression (Scale bars, 50 μm). (D) Immunofluorescence dual staining for FTO and VIMENTIN in mouse synovial tissue (Scale bars, 50 μm or 25 μm). (E) Immunofluorescence dual staining for FTO and KI67 in mouse synovial tissue (Scale bars, 50 μm or 25 μm). (F) Toluidine blue staining analysis of cartilage damage in control and AIA mice (Scale bars, 100 μm). (G) Immunofluorescence analysis of cartilage COII expression (Scale bars, 50 μm). (H) Immunohistochemistry demonstrating cartilage MMP13 expression (Scale bars, 50 μm). Data are shown as mean ± SD (n = 6). **P < 0.05, ***P < 0.01.

Having demonstrated the anti-inflammatory therapeutic effects of FTO at the cellular level, we conducted relevant animal experiments using AIA mice. We tried treating arthritis by administering FB23 through intraperitoneal injection. HE staining revealed improved therapeutic effects in the synovial tissues of AIA mice after 4 and 8 weeks of treatment, characterized by reduced synovial hyperplasia and cellular infiltration. The synovitis scores in AIA mice significantly decreased (Figure 4A). Western blot analysis indicated reduced FTO expression in the synovial tissues of AIA mice after FB23 treatment (Figure 4B). This observation was corroborated by immunohistochemistry (Figure 4C). Subsequent VIMENTIN-FTO staining demonstrated decreased FTO-positive synovial cells in the synovial tissues of AIA mice following FB23 treatment (Figure 4D). Fluorescent double staining of VIMENTIN and KI67 revealed diminished proliferative capacity in the RA mouse model after FB23 treatment (Figure 4E).

Furthermore, we assessed the condition of AIA mice articular cartilage tissues after FB23 treatment. Toluidine blue staining showed that in AIA mice treated with FB23, the cartilage layer appeared more intact, indicating an improvement in the degree of cartilage tissue damage (Figure 4F). Immunofluorescence staining for COL II revealed increased collagen content in the articular cartilage cells of treated AIA mice compared to the untreated group (Figure 4G). Immunohistochemistry demonstrated reduced expression of MMP13 in cartilage, suggesting suppression of cartilage degradation in the RA mouse model following treatment (Figure 4H). These results demonstrate the effectiveness of the treatment and are presented in Figure 4.

### RNA Seq sequencing shows that FTO can regulate CMPK2

**Figure 5:**
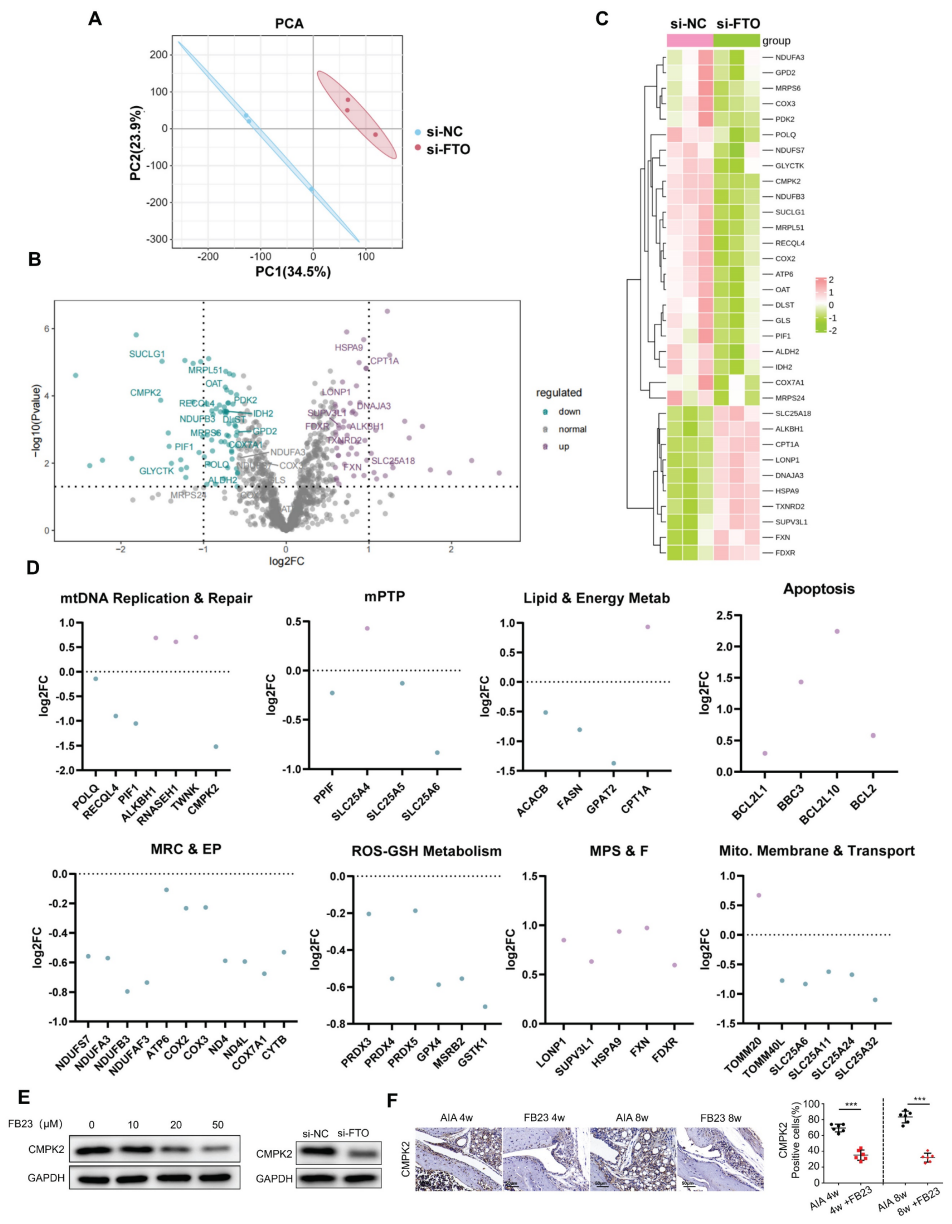
** RNA Seq sequencing shows that FTO can regulate CMPK2.** (A) Principal Component plot showing transcriptome similarity trajectories post si-NC and si-FTO interventions. Blue represents si-NC, while red represents si-FTO treatments. (B) Volcano plot displaying changes in gene transcription levels due to si-NC and si-FTO treatments. Upregulated genes are marked in purple, and downregulated genes are in blue. (C) Hierarchical clustering heat map providing further insights. (D) Quantitative representation of relative mRNA expression levels by Gene Ontology categories. Blue and purple scatter plots indicate downregulation and upregulation, respectively, following si-FTO treatment compared to si-NC. Significant changes were determined using a fold change >1.5 and p-value <0.05. (E) Western blot analysis of CMPK2 protein expression following FTO reduction. (F) Immunohistochemical staining for CMPK2 protein expression in synovial tissue of the FB23 treatment group (Scale bars, 50 μm). Data are shown as mean ± SD (n = 6). **P < 0.05, ***P < 0.01.

In order to dissect the intricate and dynamically evolving processes concerning mitochondrial stability that are orchestrated by FTO, we conducted a comprehensive transcriptomic analysis. Our analytical methodology involved the categorization of Gene Ontology (GO) terms, which are pertinent to mitochondrial functions, into distinct but interconnected groups. These categories encompassed Mitophagy, Mitochondrial Permeability Transition Pore (mPTP), mtDNA Maintenance and Repair, Mitochondrial Respiratory Chain and Energy Production (MRC & EP), Mitochondrial Protein Stability and Folding (MPS & F), Lipid and Energy Metabolism (Lipid & Energy Metab), ROS-GSH Metabolism, Apoptosis, and Mitochondrial Membrane and Transport (Mito. Membrane & Transport). Through a meticulous analysis, we identified genes exhibiting significant changes in expression patterns between si-NC and si-FTO-treated samples (Figure 5A-B).

Subsequently, we aligned these genes with a mitochondrial gene database, handpicking 1071 genes that are directly associated with mitochondrial function for further investigation. Out of these, 128 genes manifested substantial expression alterations in response to si-FTO treatment, as delineated in (Figure 5C). To delve deeper into the salient trends associated with mitochondrial stability, we conducted a detailed GO analysis, the outcomes of which are graphically illustrated in (Figure 5D). Our findings illuminated that, in cells subjected to si-FTO treatment, there was a downregulation in the expression of genes associated with mtDNA maintenance and repair, mPTP, mitochondrial respiratory chain and energy production, lipid and energy metabolism, as well as ROS-GSH metabolism (as depicted by the blue scatterplot). Conversely, there was an upregulation in gene expression related to cell apoptosis and cycle regulation, along with mitochondrial protein stability and folding (illustrated by the purple scatterplot). Following the trajectories elucidated in the preliminary phases of our analysis, we zeroed in on the potential role of CMPK2 downregulation in mitigating mtDNA leakage in si-FTO-treated cells.

Additionally, we employed Western blot analysis to assess the impact of FB23 treatment and FTO knockdown on CMPK2 expression. Our observations revealed that CMPK2 expression underwent inhibition subsequent to FTO reduction (Figure 5E). In alignment with the animal experiments detailed in Section 4, we made the noteworthy observation that AIA mice subjected to FB23 treatment exhibited diminished CMPK2 expression levels within their synovial tissues (Figure 5F).

### CMPK2 regulates inflammation through mtDNA in RA-FLS

**Figure 6:**
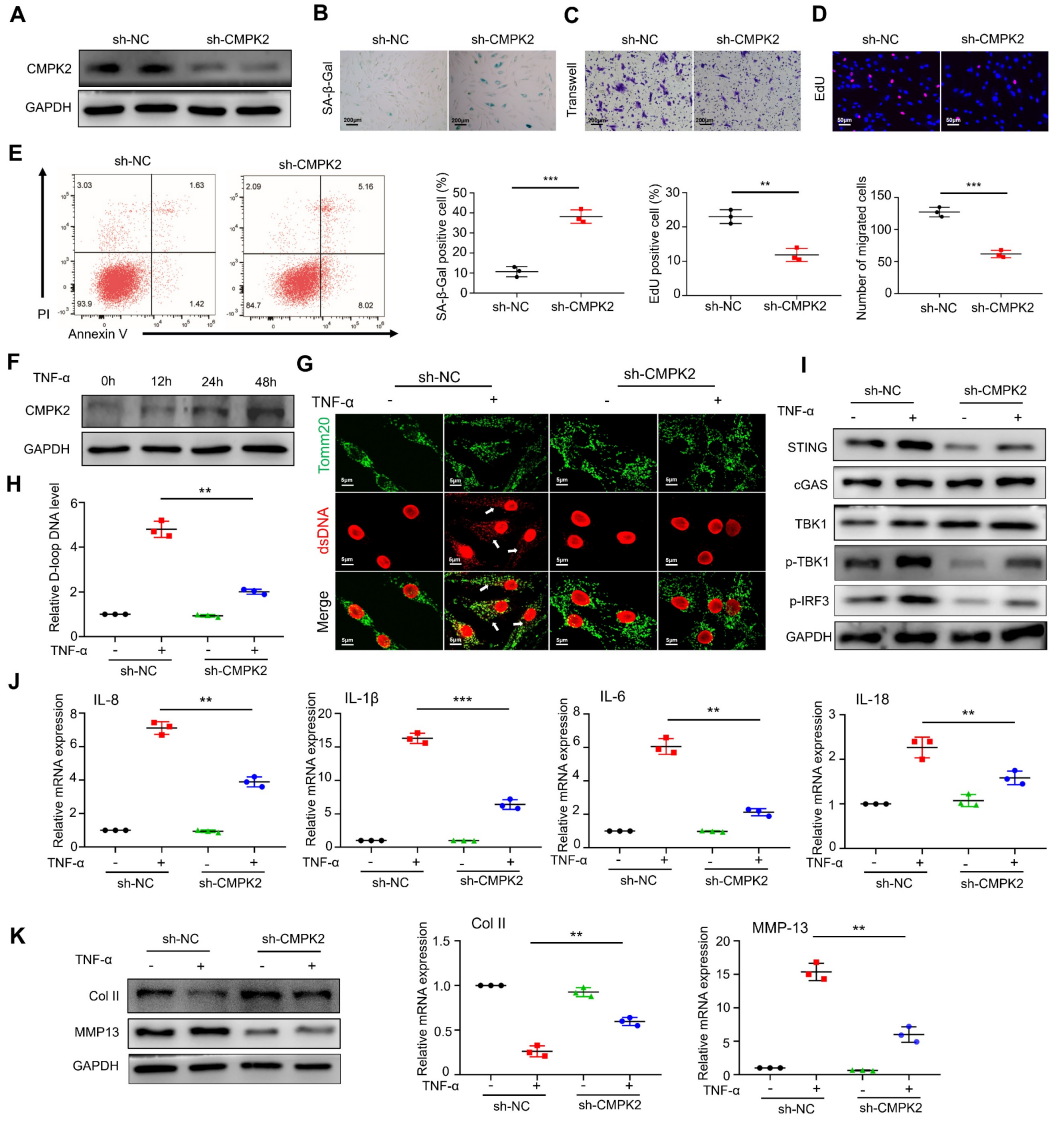
** CMPK2 regulates inflammation through mtDNA in RA-FLS.** (A) Efficiency of CMPK2 silencing in RA-FLS assessed by Western blot. (B) Impact of CMPK2 silencing on senescence in RA-FLS evaluated by β-galactosidase staining (Scale bars, 200 μm). (C) Effects of CMPK2 silencing on the migration of RA-FLS analyzed by Transwell assay (Scale bars, 200 μm). (D) Influence of CMPK2 silencing on proliferation of RA-FLS assessed by EdU staining (Scale bars, 50 μm). (E) Assessment of apoptosis in CMPK2 Knockdown RA-FLS using flow cytometry. (F) Influence of TNF-α stimulation on CMPK2 protein expression at different time points. (G) Immunofluorescence analysis of ds-DNA and Tomm20 expression in CMPK2 Knockdown RA-FLS with or without TNF-α stimulation (Scale bars, 5 μm). (H) Expression levels of D-loop DNA. (I) Activation of the cGAS/STING pathway evaluated by Western blot. (J) Expression of cytokines assessed by qPCR. (K) Expression levels of cartilage COL II and MMP13 were assessed using Western blot and qPCR, with results representative of three separate experiments. Data are shown as mean ± SD (n = 3). **P < 0.05, ***P < 0.01. ns > 0.05.

Based on the sequencing results, we hypothesized that FTO might be involved in mtDNA regulation through CMPK2. Firstly, we constructed an adenovirus for CMPK2 knockdown and confirmed a reduction in CMPK2 protein expression in RA-FLS through Western blot analysis (Figure 6A). After knocking down CMPK2, we proceeded to evaluate its impact on various cellular processes in RA-FLS, including senescence (Figure 6B), migration (Figure 6C), proliferation (Figure 6D), and apoptosis (Figure 6E). The results indicated that CMPK2 knockdown led to noticeable promotion of senescence, inhibition of proliferation, decreased migration capacity, and altered apoptosis in RA-FLS.

Next, we investigated the influence of TNF-α on CMPK2 expression and found that as the stimulation time with TNF-α increased, CMPK2 expression in RA-FLS gradually elevated, suggesting a potential role for CMPK2 in inflammation regulation (Figure 6F). Subsequently, we conducted experiments involving the addition of TNF-α on CMPK2 knockdown RA-FLS. Immunofluorescence observations revealed that in the absence of CMPK2 knockdown, TNF-α significantly induced mtDNA expression in RA-FLS, while CMPK2 knockdown resulted in reduced mtDNA expression induced by TNF-α (Figure 6G). Detection of mtDNA capability through D-loop PCR showed a decrease in mtDNA capacity in TNF-α-induced RA-FLS upon CMPK2 knockdown (Figure 6H).

Furthermore, we performed relevant tests on the mtDNA-dependent pathway, the cGAS-STING pathway. Western blot analysis demonstrated that, without CMPK2 knockdown, TNF-α induced cGAS-STING activation, while this activation was inhibited after CMPK2 knockdown (Figure 6I). Simultaneously, we examined the expression of common downstream cytokines of cGAS-STING, including IL-6, IL-8, IL-1b, and IL-18, through qPCR, revealing reduced expression of inflammatory factors after CMPK2 knockdown (Figure 6J).

Lastly, we co-cultured RA-FLS with chondrocytes in the presence of TNF-α after CMPK2 knockdown. We found that the conditioned medium from CMPK2-knockdown RA-FLS significantly reduced the damaging effects on chondrocytes. Western blot and qPCR analyses of chondrocytes indicated that co-culturing with the conditioned medium from CMPK2-knockdown RA-FLS did not significantly decrease col II levels or increase MMP13 levels (Figure 6K).

### Inhibition of CMPK2 and inflammation alleviates AIA mice arthritis progression

**Figure 7:**
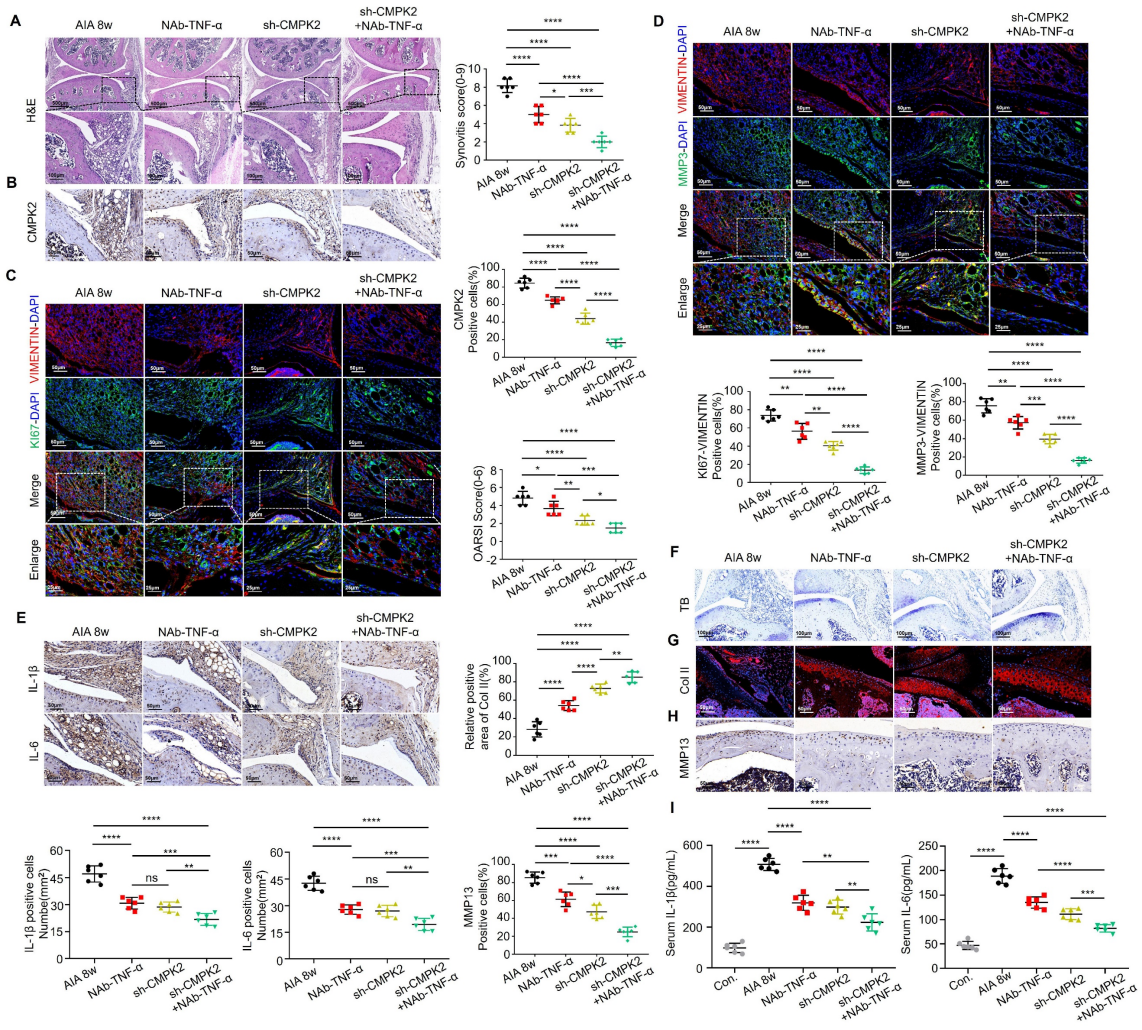
** Inhibition of CMPK2 and inflammation alleviates AIA mice arthritis progression.** (A) Representative H&E staining images: Synovium from AIA control group, TNF-α monoclonal antibody group, CMPK2 knockdown group, and CMPK2 knockdown combined with TNF-α monoclonal antibody group, with quantified arthritis scores (Scale bars, 500 or 100μm). (B) Representative immunohistochemical staining images: Synovium from AIA control group, TNF-α monoclonal antibody group, CMPK2 knockdown group, and CMPK2 knockdown combined with TNF-α monoclonal antibody group (Scale bars, 50 μm). (C) Exemplary immunofluorescence staining images along with a quantitative analysis are provided for the co-localization and scoring of Vimentin and KI67 within the synovium of each respective group (Scale bars, 50 μm or 25 μm). (D) illustrative immunofluorescence staining images and the corresponding quantitative analysis for Vimentin and MMP3 co-localization and scoring in the synovium of each experimental group (Scale bars, 50 μm or 25 μm). (E) Immunohistochemical staining detects IL-1β and IL-6 expression in synovial tissue from each group of mice (Scale bars, 50 μm). (F) Toluidine Blue staining analysis of cartilage damage in AIA and FB23-treated mice (Scale bars, 100 μm). (G) Immunofluorescence analysis of cartilage COL II expression in each group of mice (Scale bars, 50 μm). (H) Immunohistochemical analysis of MMP13 expression in cartilage of AIA and FB23-treated mice (Scale bars, 50 μm). (I) ELISA assay demonstrates reduced IL-1β and IL-6 in AIA mice serum. Data are shown as mean ± SD (n = 6). *P < 0.05, **P < 0.01, ***P < 0.001, ****P < 0.01. ns > 0.05.

To further investigate the impact of inhibiting CMPK2 and inflammation, especially TNF-α, on the progression of RA, we conducted a series of treatments in AIA mice. These interventions included weekly intraperitoneal injections of TNF-α neutralizing antibodies (NAb-TNF-α), intra-articular injections of adenovirus for CMPK2 knockdown, and combined therapies, spanning an 8-week period. Comparative analysis revealed that, in contrast to the RA model group, mice subjected to intraperitoneal NAb-TNF-α injections, intra-articular CMPK2 knockdown adenovirus injections, or a combination of both exhibited notable improvements in synovial tissue hyperplasia, reduced infiltration of inflammatory cells, and lower synovial inflammation scores. Notably, the group receiving combined treatment showed the most significant effects (Figure 7A).

Immunohistochemical staining demonstrated a consistent decrease in CMPK2 protein expression within synovial tissues across all treatment groups, with the combined treatment group displaying the most substantial reduction (Figure 7B). We further evaluated the proliferative capacity of synovial cells in treated mice through immunofluorescence double staining for VIMENTIN-KI67. The results revealed varying degrees of diminished proliferation in synovial tissues following treatment (Figure 7C). Additionally, we assessed changes in Matrix Metalloproteinase-3 (MMP3) levels within the treatment groups. MMP3 is a well-established predictor of RA disease activity and a key proteinase responsible for cartilage degradation^11^, ^12^. It also serves as a systemic inflammatory marker and can be indicative of synovial damage and prognosis in rheumatoid arthritis patients. Our findings indicated that all treatment groups led to reduced MMP3 expression in synovial tissues and a decreased number of cells co-localizing with VIMENTIN, indicating significant improvements in synovial proliferation and invasion capacity. Among these groups, the combined treatment group exhibited the most pronounced effects (Figure 7D). Immunohistochemistry confirmed that NAb-TNF-α and CMPK2 knockdown adenovirus treatment effectively reduced IL-1β and IL-6 levels in the synovium. The combined therapy demonstrated a more pronounced therapeutic effect (Figure 7E).

Subsequently, we evaluated the condition of joint cartilage in the various treatment groups. Toluidine blue staining revealed varying degrees of cartilage tissue improvement in AIA mice treated with NAb-TNF-α, CMPK2 knockdown adenovirus, or the combination of both, with the combined treatment group showing the most significant improvement (Figure 7F). Immunofluorescence staining for COL II indicated increased collagen content in joint cartilage cells of treated AIA mice compared to untreated mice (Figure 7G). Immunohistochemical staining demonstrated reduced expression of cartilage MMP13, suggesting that reducing CMPK2 and implementing anti-inflammatory treatments effectively inhibited cartilage degradation, thus mitigating the progression of RA (Figure 7H). To evaluate the systemic inflammatory response in AIA mice, we also examined the expression of IL-1β and IL-6 in the serum of AIA mice using ELISA. ELISA assays on AIA mice serum indicated that NAb-TNF-α and CMPK2 knockdown adenovirus treatment effectively suppressed the elevation of IL-1β and IL-6. The synergistic effect of the combined treatment was significantly enhanced (Figure 7I).

## Discussion

RA-FLS have a crucial role in driving the pathogenesis of RA. They contribute to sustained joint cartilage damage through aberrant proliferation, invasive behavior, and excessive inflammatory responses^13^. However, effective interventions to treat or reverse these pathological processes initiated by RA-FLS remain elusive to date^14^, ^15^. Recent studies have suggested that the enzyme FTO plays a substantial role in regulating cell proliferation and inflammation. Yet, its precise role and mechanism in RA remain unclear. The objective of this study is to explore the involvement of FTO in the advancement of RA, its potential impact on chondrocyte function, and the underlying molecular mechanisms.

FTO is a critical energy metabolism signaling protein involved in various pathophysiological processes. FTO defects lead to cell renewal impairment and premature aging^16^. Previous research has shown that knocking down FTO effectively inhibits tumor progression and attenuates cancer cell metabolism^17^. In this study, we first validated the physiological regulation of FTO in RA synovial cells, revealing that FTO is a crucial protein involved in abnormal proliferation, resistance to aging, and apoptosis in RA-FLS. Knocking down FTO alleviated the cancer-like behavior of RA-FLS. Given that rheumatoid arthritis (RA) is a chronic inflammatory condition influenced by a multitude of pro-inflammatory factors, with TNF-α as a key driver^18^ , prior research has confirmed that prolonged exposure of RA-FLS to TNF-α leads to mitochondrial dysfunction, inhibiting mitochondrial autophagy^19^, ^20^, which in turn induces and exacerbates the inflammatory process^21^, ^22^. Moreover, changes in mitochondrial DNA (mtDNA) quality are closely associated with inflammation^23^, with cytoplasmic mtDNA serving as a classic cGAS activator^24^, ^25^. The release of mtDNA into the cytoplasm activates the cGAS-STING signaling pathway, exacerbating RA disease progression^26^, ^27^. In exploring the role of TNF-α, we attempted to link FTO with inflammation.

The results revealed that FTO amplifies TNF-α-mediated inflammation, with diminished FTO resulting in reduced inflammatory capacity and suppression of TNF-α-mediated inflammation. In this process, FTO participates in the activation of the cGAS-STING pathway. After FTO knockdown, TNF-α-induced mtDNA release is reduced, and cGAS-STING pathway activation is constrained. Additionally, we investigated the regulation of downstream cytokines by cGAS-STING, primarily focusing on IL-6, IL-8, IL-1β, and IL-18^28^, ^29^. These inflammatory factors exhibit strong erosive effects on cartilage^30^ and can lead to cartilage degeneration and bone erosion^31^. Results indicated that after knocking down FTO in RA-FLS, the levels of downstream cytokines of cGAS-STING were reduced to varying degrees, attenuating the damage to chondrocytes. In this stage of research, we initially discovered that FTO participates in TNF-α-mediated mtDNA release, further regulating synovial inflammation.

FTO is intricately associated with mitochondrial biology. FTO has been shown to regulate mitochondrial thermogenesis in precursor adipocytes^32^. It also modulates mitochondrial fission/fusion and metabolism in gastric cancer cells through demethylation^33^. However, the role of FTO in RA and whether the activation of inflammatory pathways mediated by FTO is driven by other mitochondrial-related genes remain unclear. Building on this foundation, we conducted transcriptome gene analysis on RA-FLS after FTO knockdown, with a specific focus on mitochondrial-related genes, in an attempt to elucidate the association between FTO and mitochondrial-related genes. Based on transcriptomic sequencing results, we identified cytidine/uridine monophosphate kinase 2 (CMPK2) as a protein bridge involved in FTO-mediated regulation of mitochondrial function. CMPK2 is primarily localized within mitochondria and is a critical enzyme responsible for mitochondrial DNA synthesis and the maintenance of cellular UTP/CTP stability^34^. Studies have reported that CMPK2-dependent mtDNA synthesis can activate NLRP3 inflammasomes in various cell types, contributing to inflammatory responses in multiple diseases^35^, ^36^. We observed the expression of CMPK2 in RA-FLS under TNF-α stimulation and noted its significant inflammatory responsiveness. Prior research has suggested that CMPK2 deficiency leading to reduced mtDNA synthesis may result in inadequate synthesis of mtDNA-encoded proteins, subsequently leading to impaired energy production^37^, ^38^. After we established CMPK2 knockdown in RA-FLS and stimulated them with TNF-α, we examined mtDNA expression. The results confirmed that after CMPK2 knockdown, TNF-α-induced mtDNA generation and extracellular release decreased, limiting mtDNA-mediated activation of the cGAS-STING pathway and reducing downstream cytokine secretion. Having identified the crucial roles of CMPK2 and TNF-α in RA-FLS, we proceeded to validate our findings through animal experiments. We inhibited synovial cell CMPK2 while concurrently administering anti-inflammatory treatment. The results affirmed that the combined therapy more effectively suppressed the occurrence of RA synovial inflammation, consequently protecting joint cartilage. These experiments underscore that the mechanism through which CMPK2 regulates RA synovial inflammation not only directly contributes to the inflammatory control exerted by TNF-α on RA-FLS but also alters the impact of RA-FLS on joint cartilage cells' homeostasis through cytokine pathways.

However, it is imperative to acknowledge certain limitations in our research, which warrant further improvements. Due to the incomplete representation of RA patient disease states in AIA mice, additional models are needed to corroborate our findings. Furthermore, the relationship between FTO-CMPK2 expression in RA synovial tissue and the disease remains inconclusive and may necessitate larger cohorts for validation.

## Conclusion

**Figure 8:**
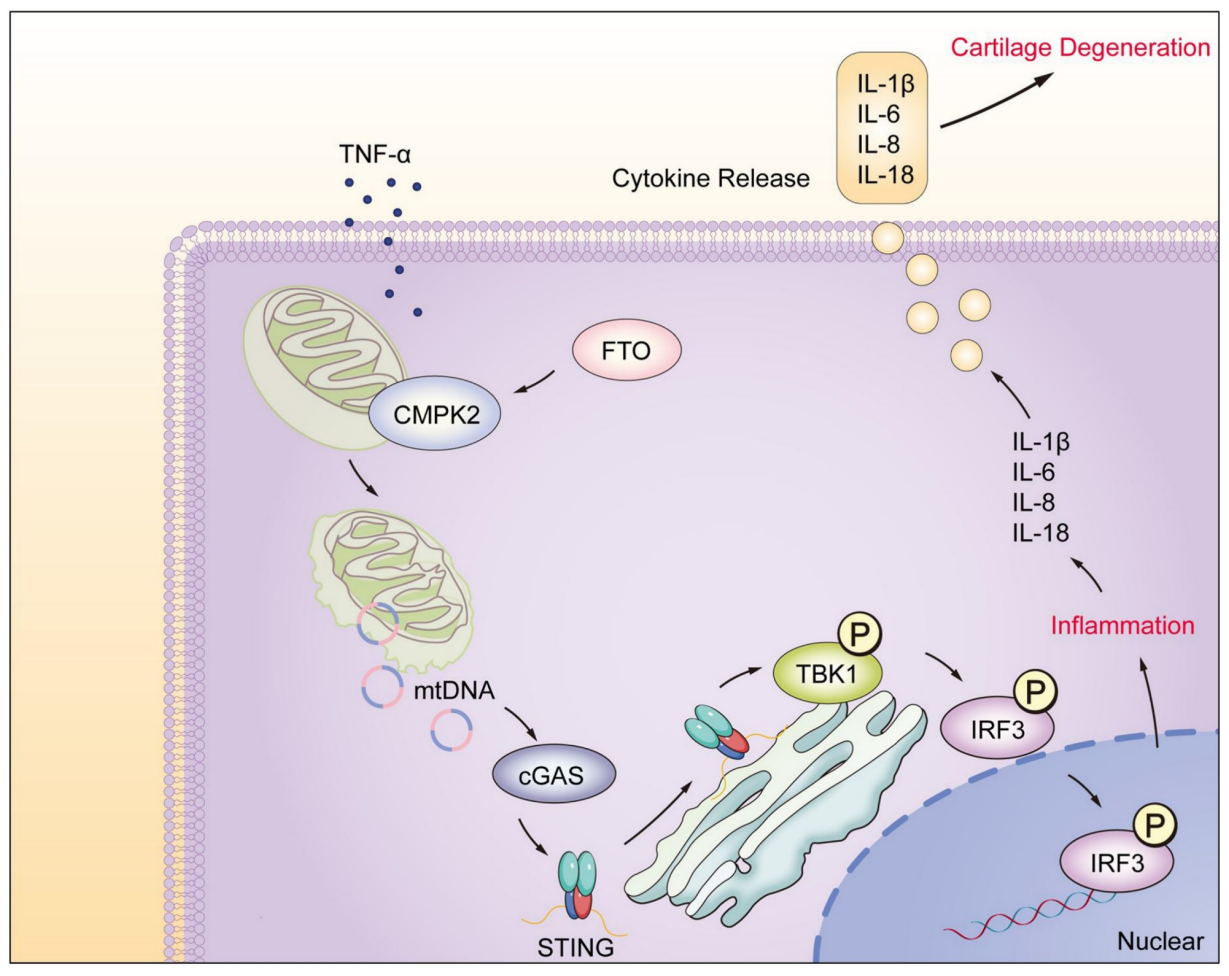
Model of RA-FLS under TNF-α stimulation, FTO-CMPK2 through mtDNA-Mediated Inflammation.

In this study, we have unveiled the significant regulatory role played by the FTO-CMPK2 pathway in rheumatoid arthritis (RA). Our investigations revealed elevated FTO expression levels within RA synovial tissues. FTO's involvement through CMPK2 in TNF-α-mediated inflammatory responses exacerbates RA synovial inflammation, ultimately leading to cartilage damage (Figure 8). Subsequent investigations have provided evidence that targeting the FTO-CMPK2 pathway and its associated inflammatory responses effectively attenuates the development of joint arthritis in AIA mice. These findings not only present novel possibilities for the treatment of RA but also emphasize the critical significance of the FTO-CMPK2 pathway.

## Figures and Tables

**Table 1:** The following primers were employed for conducting qPCR analyses to assess mRNA expression levels.

Gene	Primer forward	Primer reverse
*IL-6*	*GCCAGAGCTGTGCAGATGAG*	*GGGTCAGGGGTGGTTATTGC*
*IL-8*	*ACTCCAAACCTTTCCACCCC*	*CCCAGTTTTCCTTGGGGTCC*
*IL-1β*	*ATCTCCTGCCAA CCCTAC*	*CTTTCAGCTCATACGTGCC*
*IL-18*	*TTCGGGAAGAGGAAAGGAAC*	*AAGGATACAAAAAGTGACAT*
*MMP13*	*TCCTGATGTGGGTGAATACAAT*	*GCCATCGTGAAGTCTGGTAAAAT*
*COL II*	*TGGACGATCAGGCGAAACC*	*GCTGCGGATGCTCTCAATCT*
*GAPDH*	*TCA ACGGCACAGTCAAGG*	*ACTCCACGACATACTCAGC*

## Abbreviations

RA: rheumatoid arthritis
FLS: fibroblast-like synoviocytes
RA-FLS: rheumatoid arthritis fibroblast-like synoviocytes
FTO: fat mass and obesity-associated protein
mtDNA: mitochondrial DNA
DAMP: damage-associated molecular pattern
AIA: adjuvant-induced arthritis
OA: osteoarthritis
ACR: American College of Rheumatology
EULAR: European League Against Rheumatism
H&E: hematoxylin and eosin
TB: toluidine blue
PI: propidium iodide
mPTP: Mitochondrial Permeability Transition Pore
MRC & EP: Mitochondrial Respiratory Chain and Energy Production
Lipid & Energy Metab: Lipid and Energy Metabolism
Mito. Membrane & Transport: Mitochondrial Membrane and Transport
NAb-TNF-α: TNF-α neutralizing antibodies
